# Predicting the gastrointestinal bleeding of HBV-related acute-on-chronic liver failure based on machine learning

**DOI:** 10.3389/fmed.2025.1516476

**Published:** 2025-11-25

**Authors:** Jiwei Fu, Ahao Wu, Ziwei Zhou, Ting Deng, Pei Shi, Wentao Zhu, Mengyu Tao, Yuyu Zeng, Yuchen Peng, Yuna Wang, Xiaoping Wu

**Affiliations:** 1 Department of Infectious Diseases, The First Affiliated Hospital, Jiangxi Medical College, Nanchang University, Nanchang, Jiangxi, China; 2 Department of Digestive Surgery, Digestive Disease Hospital, The First Affiliated Hospital of Nanchang University, Nanchang, Jiangxi, China; 3 Second Department of Cardiovascular Medicine, Jiangxi Provincial People’s Hospital, The First Affiliated Hospital of Nanchang Medical College, Nanchang, Jiangxi, China

**Keywords:** chronic hepatitis B, acute-on-chronic liver failure, gastrointestinal bleeding, machine learning, survival

## Abstract

**Background:**

This study aimed to investigate the effect of gastrointestinal bleeding (GIB) on the short-term survival of hepatitis B virus-related acute-on-chronic liver failure (HBV-ACLF) patients, establish a prediction model for HBV-ACLF-related GIB via machine learning (ML) algorithms, and compare the predictive ability of various models.

**Methods:**

A total of 583 HBV-ACLF patients from two medical centers were retrospectively enrolled, and patients from one of the centers were randomly divided into a training cohort (*n* = 360) and a test cohort (*n* = 153) at a 7:3 ratio. Patients from the other center composed the validation cohort (*n* = 70). Patients were divided into GIB and non-gastrointestinal bleeding (NGIB) groups according to whether they had GIB during hospitalization, and short-term survival rates were compared between the two groups. Least absolute shrinkage and selection operator (LASSO) regression was used to screen for features associated with GIB. On the basis of the screened features, we used five ML algorithms, namely, logistic regression (LR), support vector machine (SVM), decision tree (DT), random forest (RF), and K-nearest neighbors (KNN), to build a prediction model for GIB. Six metrics, namely, accuracy, area under the curve (AUC), sensitivity, specificity, positive predictive value (PPV), and negative predictive value (NPV), were used to evaluate the predictive ability of these models.

**Results:**

In the training cohort, patients in the GIB group had significantly lower 30- and 90-day survival rates than did those in the NGIB group (48.72% versus 85.67% and 10.26% versus 64.80%, respectively), and similar results were obtained in the test cohort and the validation cohort. LASSO regression screened seven features associated with GIB, of which portal hypertension, electrolyte disturbance, and white blood cell counts were modeled features common to the five machine prediction models. The AUCs of the LR, SVM, DT, RF, and KNN models in the training cohort were 0.819, 0.924, 0.661, 1.000, and 0.865, respectively. Compared with the other four models, the LR model had the lowest PPV of 0.202 in the test cohort, the SVM model had the lowest AUC and sensitivity of 0.657 and 0.500 in the validation cohort, the DT model had the lowest sensitivity of 0.436 and 0.438 in the training and test cohorts, respectively, and the KNN model had the lowest PPV of 0.250 in the validation cohort. Notably, the RF model had the least fluctuations in accuracy, AUC, sensitivity, specificity, PPV, and NPV among the 3 cohorts, with good overall predictive ability.

**Conclusion:**

GIB has a significant effect on short-term survival in patients with HBV-ACLF. On this basis, five ML prediction models, LR, SVM, DT, RF, and KNN, were established to have better prediction ability for GIB, among which the RF model has the most robust prediction performance, which can help clinicians intervene in advance and improve the short-term survival rate of patients.

## 1 Introduction

Acute-on-chronic liver failure (ACLF) is a severe clinical syndrome resulting from acute exacerbation of chronic liver disease and is characterized by a dramatic decline in liver function, multi-organ failure, and a high mortality rate (1). Hepatitis B virus (HBV) infection is the leading cause of ACLF in Asian populations, accounting for approximately 70% of the cases (2). Hepatitis B virus-related acute–chronic liver failure (HBV-ACLF) has a high mortality rate of approximately 54.3% (3). Various complications, such as gastrointestinal bleeding (GIB), hepatic encephalopathy, and hepatorenal syndrome, are some of the main reasons for the high mortality rate of HBV-ACLF. However, there is still some controversy as to whether GIB affects the survival of ACLF patients (4).

GIB is a relatively common complication of HBV-ACLF (5). The prevalence of GIB in patients with HBV-ACLF is approximately 13.86% (6). A study from China reported that upper GIB has no significant effect on short-term survival in patients with ACLF (7). However, another study found that among ACLF patients with complicated variceal bleeding, the short-term risk of death increased (8). Guo et al. compared the prognostic impact of combined bleeding in patients with HBV-ACLF and reported that the survival rate in the bleeding group (51.85%) was lower than that in the nonbleeding group (78.33%) (6). Suppose the effect of GIB on the survival of HBV-ACLF patients can be clarified. In these cases, the related risk factors for its occurrence can be identified, and GIB events can be predicted in advance; thus, intervention can effectively reduce the mortality rate of HBV-ACLF patients. Currently, there are few studies on the prediction model of GIB occurrence in HBV-ACLF patients, and only a few related studies (6, 7, 9), such as those regarding the prediction model of esophagogastric variceal bleeding in patients with chronic liver failure, the prediction model of bleeding in HBV-ACLF patients, and upper GIB in patients with ACLF, have the problems of fewer research subjects and no external data validation, which make it difficult to accurately predict the occurrence of GIB events in patients with HBV-ACLF. Therefore, a new model that can predict the occurrence of GIB in patients with HBV-ACLF is urgently needed.

Machine learning (ML) has considerable advantages in diagnosing and predicting diseases. With the rapid, recent development of artificial intelligence, its integration with clinical practice has become increasingly intense, and has also been widely used in the field of liver failure (10). For example, a support vector machine (SVM) was used to construct a 28-day prognostic model for ACLF (11), predict the prognosis of liver transplantation for ACLF via a decision tree (DT) model (12), predict the prognostic status of acetaminophen-induced acute liver failure via a random forest (RF) model (13), construct a predictive model for liver failure after hepatic cancer resection using logistic regression (LR) (14), and apply the k-nearest neighbors (KNN) algorithm to construct a biomolecular mapping model of albumin to identify acute liver failure severity (15). Although the development of ML algorithms has significantly improved the accuracy of predictive models in diagnosing liver failure and improving survival prognosis, there are differences in predictive efficacy when different ML algorithms are used for modeling (16). Therefore, choosing the appropriate ML predictive model requires further comparative analysis.

This study aimed to investigate the effect of GIB on the survival of patients with HBV-ACLF. Additionally, for the first time, ML algorithms were used to construct a prediction model for concurrent GIB in HBV-ACLF patients, and the model was validated with internal and external data. Interfering in advance for HBV-ACLF patients at high risk of GIB, reducing the mortality of ACLF patients, and providing a theoretical basis for clinical work are needed.

## 2 Materials and methods

### 2.1 Study population and design

**Figure 1:**
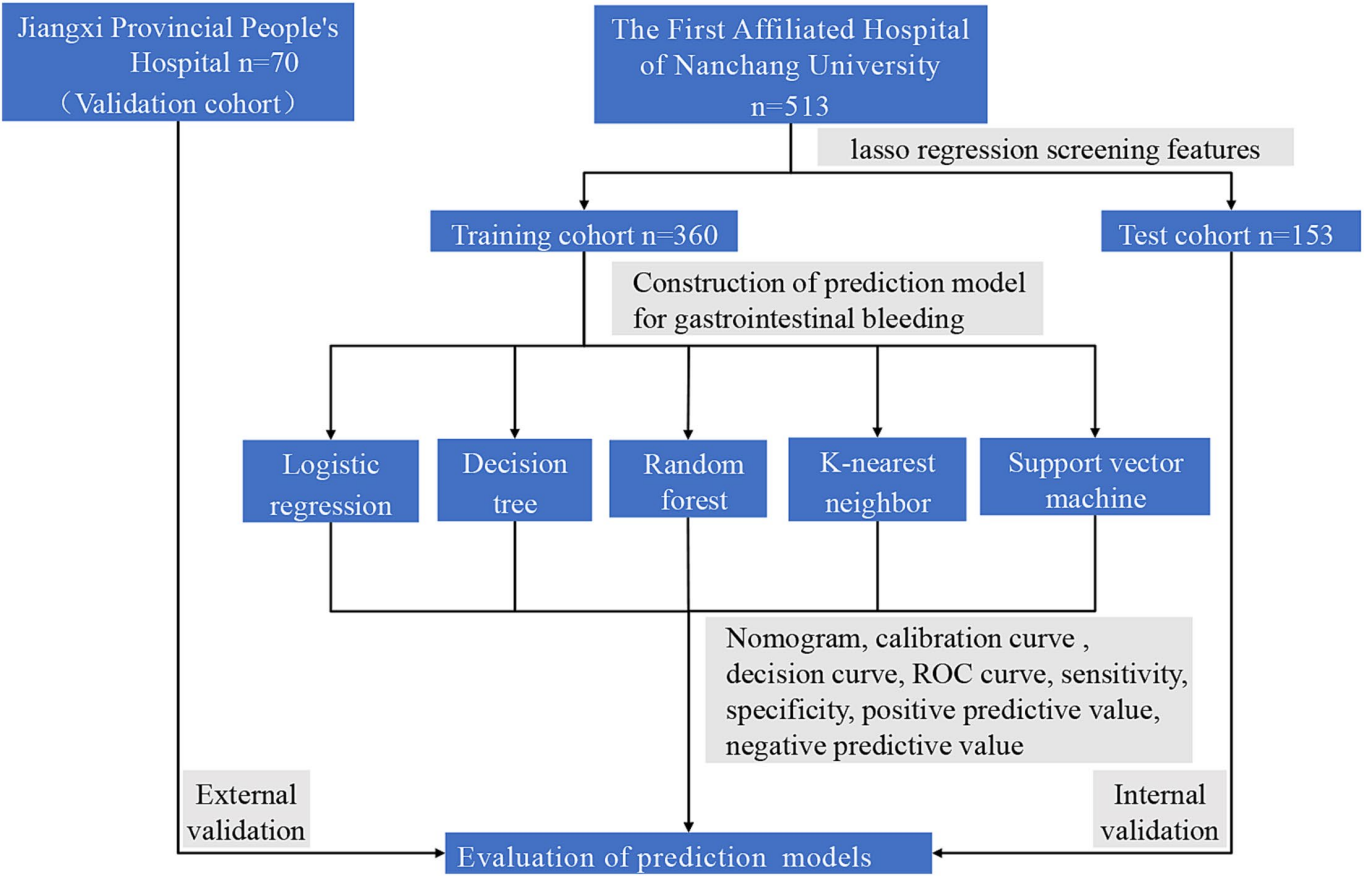
Flowchart of the study.

Data on 583 HBV-ACLF patients hospitalized at the Department of Infection of the First Affiliated Hospital of Nanchang University and Jiangxi Provincial People’s Hospital from 2014 to 2021 were retrospectively collected. Among them, 513 patients from the First Affiliated Hospital of Nanchang University were randomly divided into a training cohort (*n* = 360) and a test cohort (*n* = 153) at a ratio of 7:3. Additionally, 70 patients from Jiangxi Provincial People’s Hospital were used as a validation cohort to externally validate the model. The patients were divided into a GIB group and an NGIB group according to whether they had GIB. The study flow is detailed in Figure 1. Inclusion criteria were as follows: (1) patients were collected on the basis of the diagnostic criteria of the Asian Pacific Association for the Study of the Liver (17): serum bilirubin ≥5 mg/dL, international normalized ratio (INR) ≥ 1.5 or prothrombin activity (PTA) < 40% based on chronic liver disease or cirrhosis and ascites or hepatic encephalopathy within 4 weeks; and (2) serum HBV surface antigen (HBsAg) positivity lasting more than 6 months. The exclusion criteria were as follows: (1) infection with other hepatotropic viruses (ex. Hepatitis A, C, E); (2) combined malignant tumor of the liver; (3) other severe diseases, such as respiratory/heart failure or end-stage renal disease; and (4) missing data. The Medical Research Ethics Board of the First Affiliated Hospital of Nanchang University, China, waived informed consent in the main manuscript (Ethics Board approval number: (IIT [2021]100)). All research methods were conducted in accordance with the principles of the Declaration of Helsinki.

### 2.2 Data collection

The clinical demographic records (sex, age, weight, etc.), past medical history (hypertension, diabetes, cirrhosis), laboratory tests (routine blood tests, liver and kidney function, glucose, lipids, coagulation indicators, etc.), complications before GIB (hepatic encephalopathy, hepatorenal syndrome, ascites, spontaneous bacterial peritonitis, electrolyte disturbance), radiographic examinations, treatment programs, and 30- and 90-day survival rates were collected. GIB was diagnosed on the basis of the presence of vomited blood or dark stools combined with a positive occult blood test during the patient’s hospitalization.

Artificial liver refers to the use of medical devices or technologies to simulate or replace part of the liver’s function, alleviating clinical symptoms in patients with liver failure or severe liver diseases. Common methods of artificial liver treatment include Hemodialysis, Plasma Exchange/Adsorption, and Biological Artificial Liver. Currently, artificial liver therapy is mainly used in the treatment of various types of liver failure, such as acute liver failure, ACLF, and liver dysfunction (18).

### 2.3 SVM

SVM is a classical algorithm for classification that is used to address binary or multiple classification problems. The essence is to find an optimal hyperplane in the feature space that allows classification to be completed while keeping the interval maximum. Small sample data are handled better, but too many variables affect its classification efficiency (19). The “e1071” R package was downloaded for constructing SVM prediction models in the R language.

### 2.4 DT and RF

A DT is a classification and regression method named for its rules and decisions that resemble the trunks and branches of a tree. The predictor variable in a DT acts as the root node, the prediction outcome acts as a leaf node, and the path connecting the two is the decision rule. The algorithm finds the optimal variables and combinations to classify the data correctly (20). RF is a collection of DTs that improves the robustness of the model and reduces overfitting situations, resulting in better predictive performance (21). We use the “part” and “randomForest” packages in R to construct the above two models.

### 2.5 KNN

KNN is a simple classification algorithm that works by calculating the distance of the variable to be classified from all neighboring training points and determining k loci, which allows the variable to be classified into a class of proximity (22). A diagnostic model for GIB was constructed using the “knn” package.

### 2.6 LR

Multivariate logistic regression analysis was performed via SPSS. The screened independent risk factors were used to construct an LR diagnostic model of the training cohort via a forward stepwise regression approach (23).

### 2.7 Statistical analysis

Statistical analysis was performed with R (version 4.13), SPSS (version 26.0), and GraphPad Prism (version 8.0). Count data are presented as frequencies, and comparisons between groups were made via the chi-square test. The measurement data were first tested for a normal distribution. Normally distributed measurement data are described by X ± S (mean standard deviation), two independent samples t tests were used for comparisons between two groups, and one-way ANOVA was used for comparisons between multiple groups. Non-normally distributed measurement data are described by M (P25–P75) (median, upper and lower quartiles), the Mann–Whitney U test was used for comparisons between two groups, and the Kruskal–Wallis H rank sum test was used for comparisons between multiple groups. The possible risk factors for GIB were subjected to LASSO regression analysis, and the filtered features were further put into a predictive model via five ML algorithms, namely, multifactorial LR, SVM, DT, RF, and KNN. The model was visualized with a nomogram. Predictive models were evaluated in terms of accuracy, area under the curve (AUC), sensitivity, specificity, positive predictive value (PPV), and negative predictive value (NPV). Finally, we performed internal and external validation of the constructed model. *p* < 0.05 indicated a statistically significant difference.

### 2.8 Ethics approval and informed consent

The study was approved by the Ethics Committee of the First Affiliated Hospital of Nanchang University (IIT [2021]100). As this study was retrospective, the Ethics Committee of the First Affiliated Hospital of Nanchang University waived the informed consent for this study. All research methods were conducted in accordance with the principles of the Declaration of Helsinki.

## 3 Results

### 3.1 Clinical characteristics

Among the 583 patients with HBV-ACLF, 63 had GIB, with a prevalence of 10.8%. Among them, 513 patients at the First Affiliated Hospital of Nanchang University were randomly divided into a training cohort (*n* = 360) and a test cohort (*n* = 153) at a ratio of 7:3. The training cohort included 311 males and 49 females, with a mean age of 44.66 ± 12.06 years, and 39 patients with GIB. The test cohort included 131 males and 22 females with a mean age of 44.48 ± 11.98 years and 16 patients with GIB. Seventy patients from Jiangxi Provincial People’s Hospital served as the validation cohort, including 59 males and 11 females, with a mean age of 44.54 ± 13.27 years, and 8 patients with GIB. A comparison of the clinical characteristics of the 3 cohorts of patients revealed no statistically significant differences (Table 1), which confirmed the reliability of the results of the test and validation cohorts.

**Table 1:** HBV-ACLF patient characteristics in the training, test, and validation cohorts.

Characteristics	Training cohort (*n* = 360)	Test cohort (*n* = 153)	Validation cohort (*n* = 70)	*p* value
Gender				0.891
Male	311	131	59	
Female	49	22	11	
Age (years)	44.66 ± 12.06	44.48 ± 11.98	44.54 ± 13.27	0.988
Weight (kg)	64.95 ± 10.37	64.17 ± 11.04	65.20 ± 11.23	0.704
Hypertension				
No	314	139	62	0.503
Yes	46	14	8	
Diabetes				1.000
No	334	142	65	
Yes	26	11	5	
Cirrhosis				0.597
No	235	100	50	
Yes	125	53	20	
Portal hypertension				0.664
No	293	127	60	
Yes	67	26	10	
Hepatic encephalopathy				0.419
No	254	114	54	
Yes	106	39	16	
Hepatorenal syndrome				0.115
No	310	129	66	
Yes	50	24	4	
Gastrointestinal bleeding				0.976
No	321	137	62	
Yes	39	16	8	
Ascites				0.160
No	156	60	22	
Yes	204	93	48	
Spontaneous bacterial peritonitis				0.120
No	148	59	37	
Yes	212	94	33	
Electrolyte disturbance				0.909
No	205	84	39	
Yes	155	69	31	
Number of artificial liver treatments	1.00 (0.00, 2.00)	1.00 (0.00, 2.00)	0.00 (0.00, 2.25)	0.328
Glucocorticoid therapy				0.796
No	212	93	44	
Yes	148	60	26	
WBC (×10^9^/L)	6.28 (4.57, 8.25)	6.15 (5.16, 8.16)	6.02 (4.53, 7.94)	0.692
HB (g/L)	130.55 ± 20.95	130.64 ± 19.57	129.21 ± 20.98	0.873
PLT (×10^9^/L)	123.13 ± 55.41	132.71 ± 55.73	126.33 ± 54.94	0.202
ALT (U/L)	761.86 ± 682.15	717.38 ± 618.34	581.34 ± 611.11	0.108
AST (U/L)	552.83 ± 564.77	523.49 ± 477.51	459.81 ± 431.00	0.388
TBIL (μmol/L)	290.65 (205.75, 380.28)	307.80 (212.40, 397.55)	259.60 (170.28, 348.15)	0.052
DBIL (μmol/L)	186.71 ± 88.81	184.03 ± 86.31	167.49 ± 112.16	0.273
ALB (g/L)	32.31 ± 4.34	32.41 ± 3.98	31.06 ± 4.38	0.057
SCR (μmol/L)	73.70 ± 33.70	77.22 ± 40.39	78.85 ± 43.25	0.418
Glu (mmol/L)	5.23 ± 3.45	5.06 ± 3.51	6.07 ± 3.13	0.110
TG (mmol/L)	1.12 ± 0.70	1.12 ± 0.71	1.16 ± 1.23	0.925
TC (mmol/L)	2.60 ± 0.96	2.50 ± 0.86	2.59 ± 1.28	0.590
PT (s)	23.20 (19.13, 30.35)	24.70 (20.20, 30.90)	24.80 (21.78, 28.98)	0.183
PTA (%)	32.35 (24.23, 41.50)	30.90 (23.00, 38.80)	36.00 (30.00, 38.00)	0.074
INR	2.11 (1.71, 2.74)	2.24 (1.83, 2.80)	2.16 (2.05, 2.50)	0.121
FIB (g/L)	1.35 ± 1.05	1.41 ± 1.80	1.82 ± 2.57	0.063
D-Dimer (mg/L)	2.72 ± 4.22	2.6 ± 3.48	2.15 ± 2.20	0.520
HBV-DNA (log10 IU/mL)	5.37 (3.63, 6.93)	5.31 (3.34, 6.92)	5.66 (1.00, 7.13)	0.831

WBC, white blood cells; HB, hemoglobin; PLT, platelets; ALT, alanine aminotransferase; AST, aspartate aminotransferase; TBIL, total bilirubin; DBIL, direct bilirubin; ALB, albumin; SCR, serum creatinine; Glu, glucose; TG, triglyceride; TC, total cholesterol; PT, prothrombin time; PTA, prothrombin activity; INR, international normalized ratio; FIB, fibrinogen; HBV-DNA, hepatitis B virus DNA.

### 3.2 Effect of GIB on short-term survival in HBV-ACLF patients

**Figure 2:**
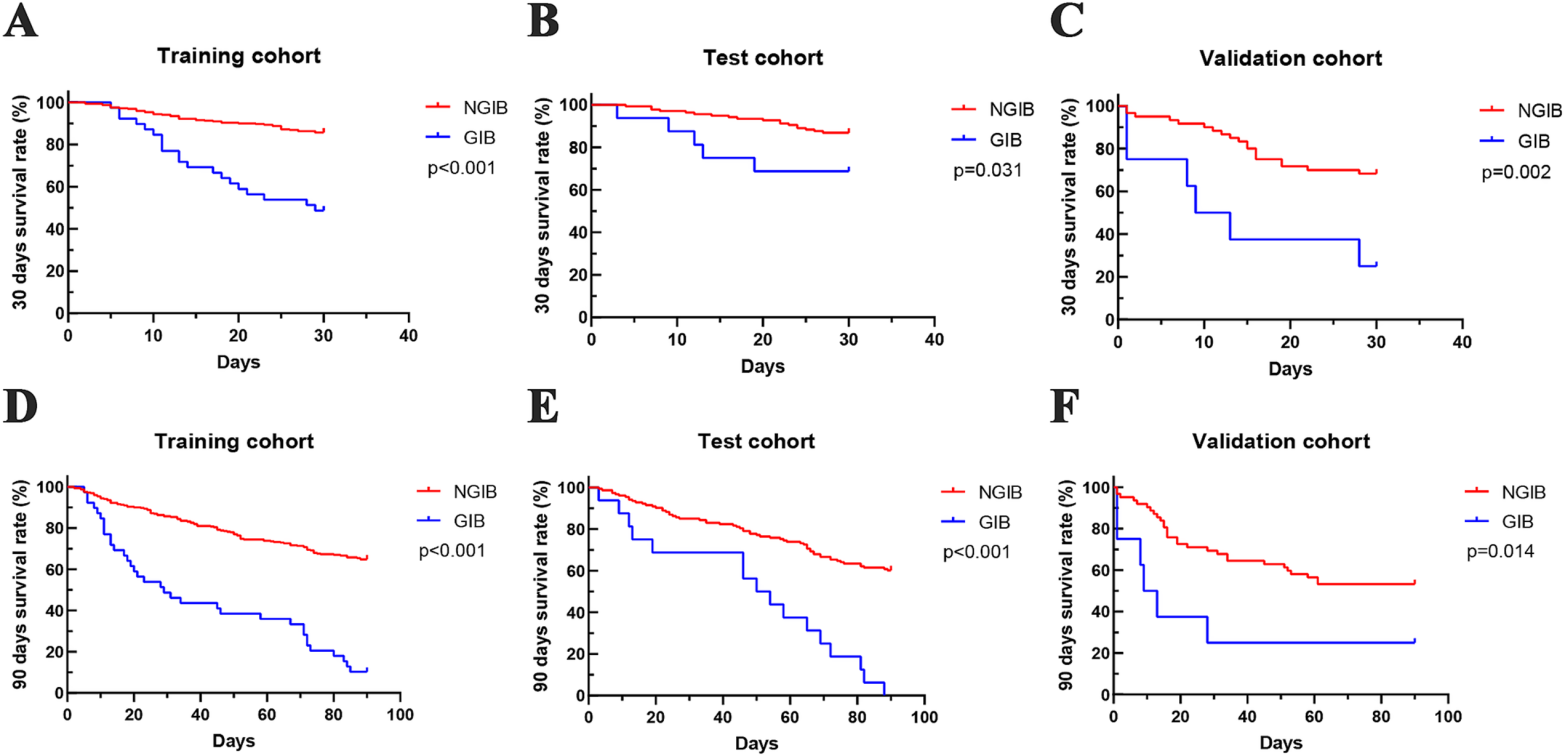
The 30-day and 90-day survival of patients with HBV-ACLF in the GIB group versus the non-GIB group. The 30-day survival curve for the **(A)** training group, **(B)** test group, and **(C)** validation group. The 90-day survival curve for the **(D)** training group, **(E)** test group and **(F)** validation group.

We compared the 30- and 90-day survival rates between the GIB group and the NGIB group in the 3 cohorts (Figures 2A–F). In the training cohort, the 30-day survival rate in the GIB group versus the NGIB group was 48.72% versus 85.67%, *p* < 0.001, and the 90-day survival rate was 10.26% versus 64.80%, *p* < 0.001. In the test cohort, the 30-day survival rate in the GIB group versus the NGIB group was 68.75% versus 86.86%, *p* = 0.031, and the 90-day survival rate was 0% versus 67.15%, *p* < 0.001, respectively. In the validation cohort, the 30-day survival rate in the GIB group versus the NGIB group was 25% versus 67.74%, *p* = 0.002, and the 90-day survival rate was 25% versus 51.61%, *p* = 0.014, respectively. In all 3 cohorts, patients in the GIB group had significantly lower 30- and 90-day survival rates than those in the NGIB group did.

### 3.3 Selection of clinical characteristics

**Figure 3:**
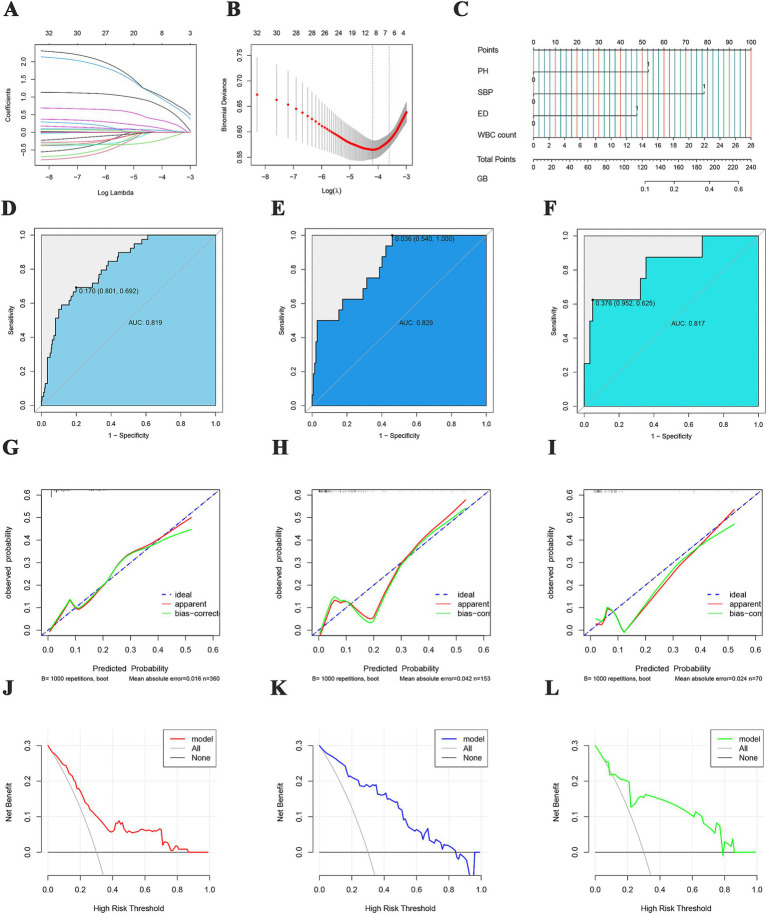
Feature screening and LR regression modeling. **(A)** Relationships between the lambda values and feature coefficients; **(B)** relationships between the lambda values and bias percentages of the features; **(C)** training cohort-based nomogram; **(D)** training cohort ROC curve; **(E)** test cohort ROC curve; **(F)** validation cohort ROC curve; **(G)** training cohort fitting curve; **(H)** test cohort fitting curve; **(I)** validation cohort fitting curve; **(J)** training cohort calibration curve; **(K)** test cohort calibration curve; **(L)** validation cohort calibration curve.

A total of 32 characteristics of HBV-ACLF patients were collected, and LASSO regression analysis was performed to analyze the above characteristics of 513 patients at the First Affiliated Hospital of Nanchang University. The larger the value of *λ* is, the closer the feature coefficients are to 0, whereas the bias percentage tends to decrease and then increase, with an optimal *λ*-se of 7 (Figures 3A,B). Seven clinical features associated with GIB, including white blood cell count, cholesterol, number of artificial liver treatments, portal hypertension, spontaneous bacterial peritonitis, hepatorenal syndrome, and electrolyte disturbance, were selected on the basis of the optimal λ value.

### 3.4 LR-based predictive model

The seven characteristics selected by LASSO regression were included in a multivariate logistic regression analysis, which revealed that portal hypertension (OR = 2.947, 95% CI 1.270–6.839, *p* = 0.012), spontaneous bacterial peritonitis (OR = 5.975, 95% CI 1.708–20.904, *p* = 0.005), electrolyte disturbance (OR = 3.300, 95% CI 1.510–7.210, *p* = 0.003) and white blood cell count (OR = 1.095, 95% CI 1.013–1.184, *p* = 0.023) were four independent risk factors for GIB (Table 2). A prediction model was constructed on the basis of the above four characteristics: Logit(P) = ln[P/(1 − P)] = 0.993 × [Portal hypertension (0 No, 1 Yes)] + 2.025 × [Spontaneous bacterial peritonitis (0 No, 1 Yes)] + 1.205 × [Electrolyte disturbance (0 No, 1 Yes)] + 0.108 × [White blood cell count (×10^9^/L)]-5.414. To visualize the model, we simultaneously constructed a nomogram (Figure 3C). In the training cohort, the AUC value of the model was 0.819, and the sensitivity, specificity, PPV, and NPV were 0.692, 0.801, 0.300, and 0.955, respectively (Figure 3D). In the test cohort, the AUC of the model was 0.829, and the sensitivity, specificity, PPV, and NPV were 1.000, 0.540, 0.202, and 1.000, respectively (Figure 3E). In the validation cohort, the AUC of the model was 0.817, and the sensitivity, specificity, PPV, and NPV were 0.625, 0.952, 0.600, and 0.952, respectively (Figure 3F). In the training cohort, the calibration curves ideally and realistically fit better than those in the test and validation cohorts did (Figures 3G–I). All three cohort decision curves showed a better clinical net benefit of the model (Figures 3J–L).

**Table 2:** Multivariate logistic regression analysis of patient characteristics associated with increased gastrointestinal bleeding risk.

Characteristics	*B*	OR	OR 95% L	OR 95% H	*p*
Number of artificial liver treatments	0.217	1.243	0.919	1.680	0.158
Portal hypertension	1.081	2.947	1.270	6.839	0.012
Spontaneous bacterial peritonitis	1.788	5.975	1.708	20.904	0.005
Hepatorenal syndrome	0.549	1.732	0.735	4.081	0.209
Electrolyte disturbance	1.194	3.300	1.510	7.210	0.003
WBC (×10^9^/L)	0.091	1.095	1.013	1.184	0.023
TG (mmol/L)	−0.337	0.714	0.456	1.118	0.141

WBC, white blood cells; TG, triglyceride.

### 3.5 SVM-based predictive model

**Figure 4:**
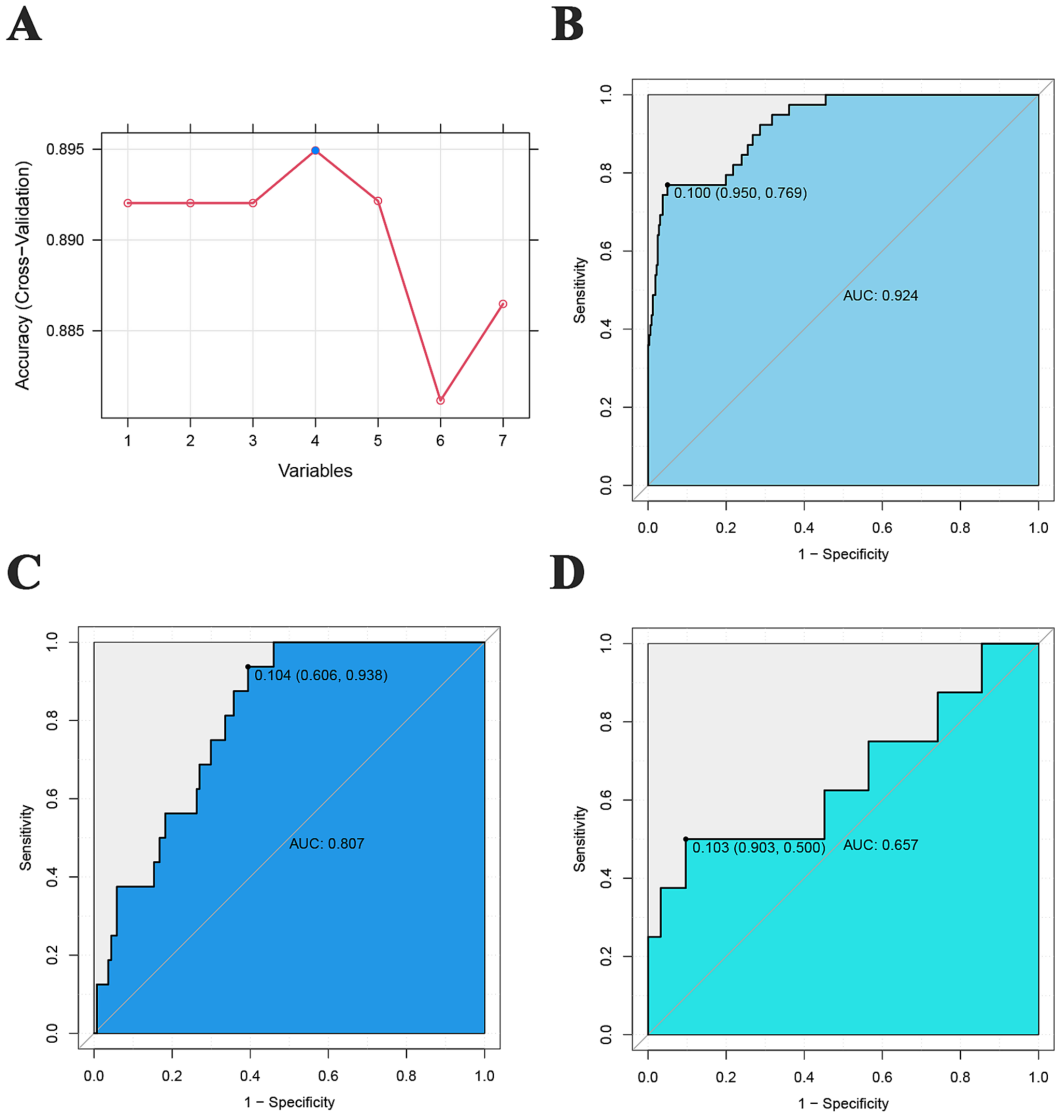
SVM prediction model. **(A)** Number of vectors and model accuracy of the SVM; **(B)** training cohort ROC curve; **(C)** test cohort ROC curve; **(D)** validation cohort ROC curve.

The GIB model constructed by SVM had the highest accuracy when the number of vectors was 4 (Figure 4A). In the training cohort, the AUC value of the model was 0.924, and the sensitivity, specificity, PPV, and NPV were 0.769, 0.950, 0.652, and 0.971, respectively (Figure 4B). In the test cohort, the AUC of the model was 0.807, and the sensitivity, specificity, PPV, and NPV were 0.938, 0.606, 0.217, and 0.895, respectively (Figure 4C). In the validation cohort, the AUC of the model was 0.657, and the sensitivity, specificity, PPV, and NPV were 0.500, 0.903, 0.400, and 0.933, respectively (Figure 4D).

### 3.6 DT-based predictive model

**Figure 5:**
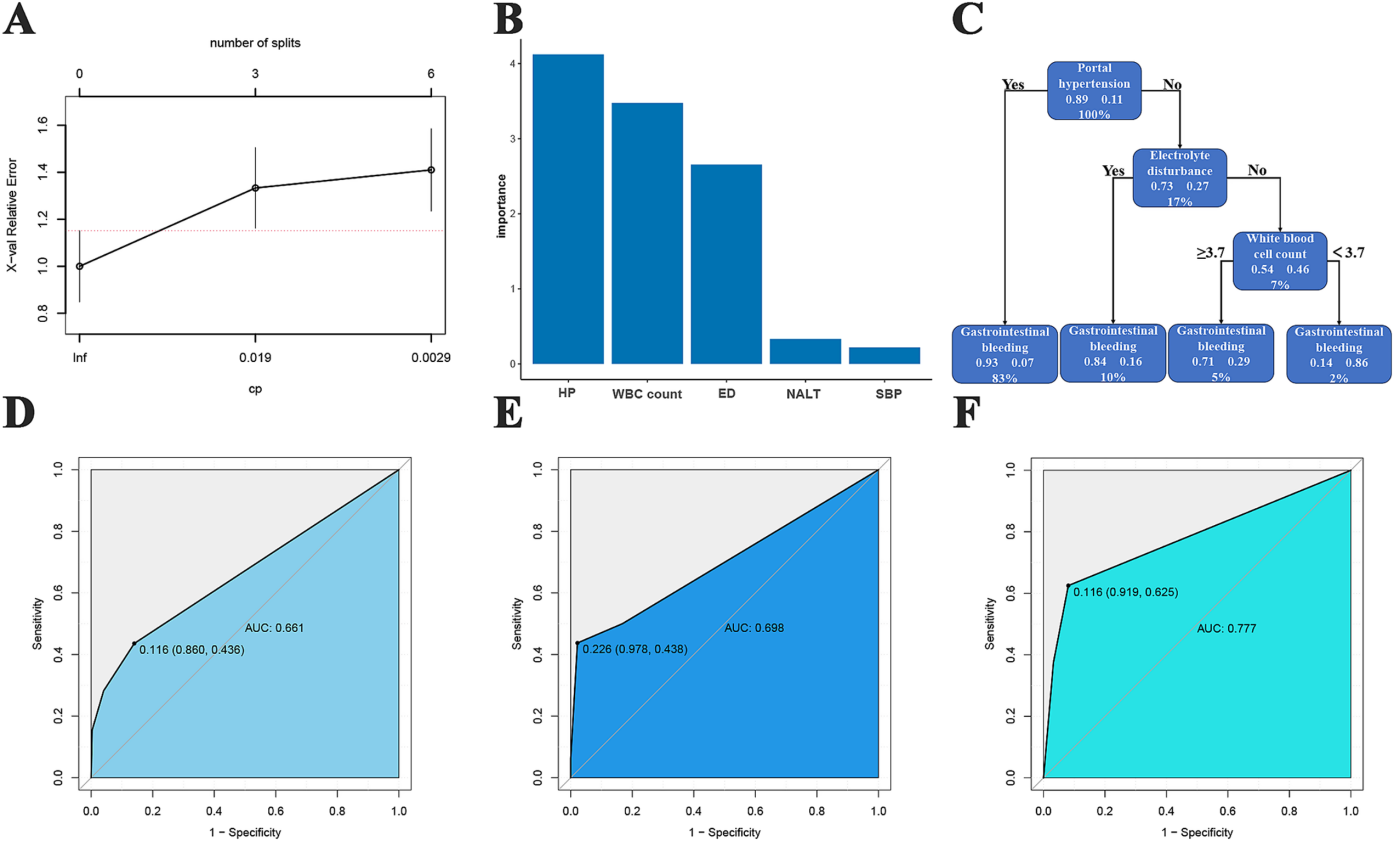
DT predictive model. **(A)** Relationships between the number of splitting points of DTs and complexity parameters; **(B)** the significance of clinical features and radiomic features for the DT-based predictive model; **(C)** decision tree diagram; **(D)** training cohort ROC curve; **(E)** test cohort ROC curve; **(F)** validation cohort ROC curve.

In the DT model, we identified the 3 best split nodes (Figure 5A). The model characteristics, in descending order of importance, were portal hypertension, white blood cell count, electrolyte disturbance, number of artificial liver treatments, and spontaneous bacterial peritonitis (Figure 5B). On this basis, the top 3 most important features were selected to construct the prediction model (Figure 5C). In the training cohort, the AUC of the model was 0.661, and the sensitivity, specificity, PPV, and NPV were 0.436, 0.86, 0.274, and 0.926, respectively (Figure 5D). In the test cohort, the AUC of the model was 0.698, and the sensitivity, specificity, PPV, and NPV were 0.438, 0.978, 0.700, and 0.937, respectively (Figure 5E). In the validation cohort, the AUC value of the model was 0.777, and the sensitivity, specificity, PPV, and NPV were 0.625, 0.919, 0.500, and 0.950, respectively (Figure 5F).

### 3.7 RF-based predictive model

**Figure 6:**
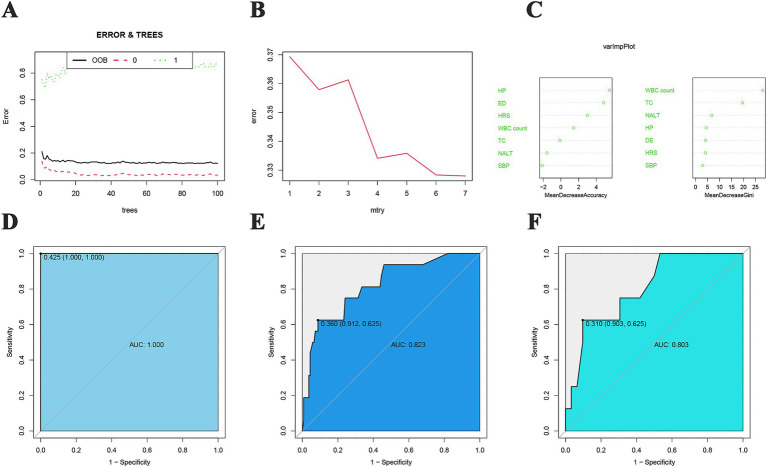
RF prediction model. **(A)** Relationship between the number of trees and OOB error in RF; “0” represents the GIB group, and “1” represents the NGIB group; **(B)** relationship between the number of split points and error in the tree; **(C)** importance of features in improving the model accuracy and reducing the Gini coefficient; **(D)** training cohort ROC curve; **(E)** test cohort ROC curve; **(F)** validation cohort ROC curve.

In the RF prediction model, the out-of-bag (OOB) error reached a minimum value of 0.122 when the number of trees in the model was 37 (Figure 6A). Furthermore, the error value was minimized to 0.328 when the split node of the tree was 7 (Figure 6B). We then analyzed the importance of the 7 features included, and the top 3 features that improved model accuracy and reduced the Gini coefficient of the model were portal hypertension, electrolyte disturbance, and hepatorenal syndrome, as well as white blood cell count, total cholesterol, and number of artificial liver treatments, respectively (Figure 6C). In the training cohort, the AUC of the model was 1.000, and the sensitivity, specificity, PPV, and NPV were 1.000, 1.000, 1.000, and 1.000, respectively (Figure 6D). In the test cohort, the AUC of the model was 0.823, and the sensitivity, specificity, PPV, and NPV were 0.625, 0.912, 0.455, and 0.954, respectively (Figure 6E). In the validation cohort, the AUC value of the model was 0.803, and the sensitivity, specificity, PPV, and NPV were 0.625, 0.903, 0.455, and 0.949, respectively (Figure 6F).

### 3.8 KNN-based predictive model

**Figure 7:**
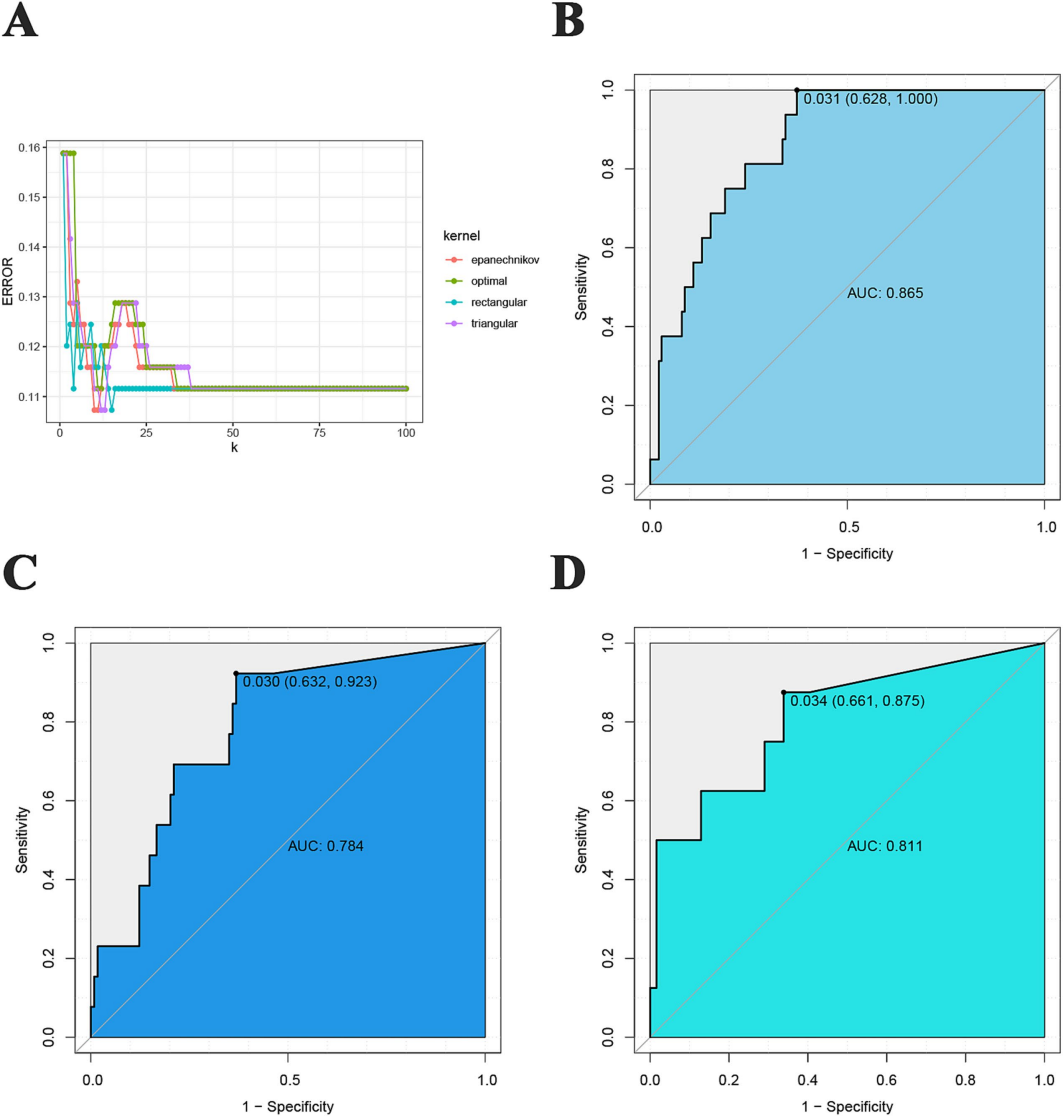
KNN prediction model. **(A)** Screening for the best kernel function and k value; **(B)** training cohort ROC curve; **(C)** test cohort ROC curve; **(D)** validation cohort ROC curve.

By performing hyperparametric optimization of the KNN function, we obtained the optimal kernel function as triangular as well as the optimal K value of 12 (Figure 7A). In the training cohort, the AUC of the model was 0.865, and the sensitivity, specificity, PPV and NPV were 1.000, 0.628, 0.247, and 1.000, respectively (Figure 7B). In the test cohort, the AUC of the model was 0.784, and the sensitivity, specificity, PPV, and NPV were 0.923, 0.632, 0.200, and 0.989, respectively (Figure 7C). In the validation cohort, the AUC value of the model was 0.811, and the sensitivity, specificity, PPV, and NPV were 0.875, 0.661, 0.250, and 0.976, respectively (Figure 7D).

### 3.9 Comparison of the predictive power of the 5 machine prediction models

**Figure 8:**
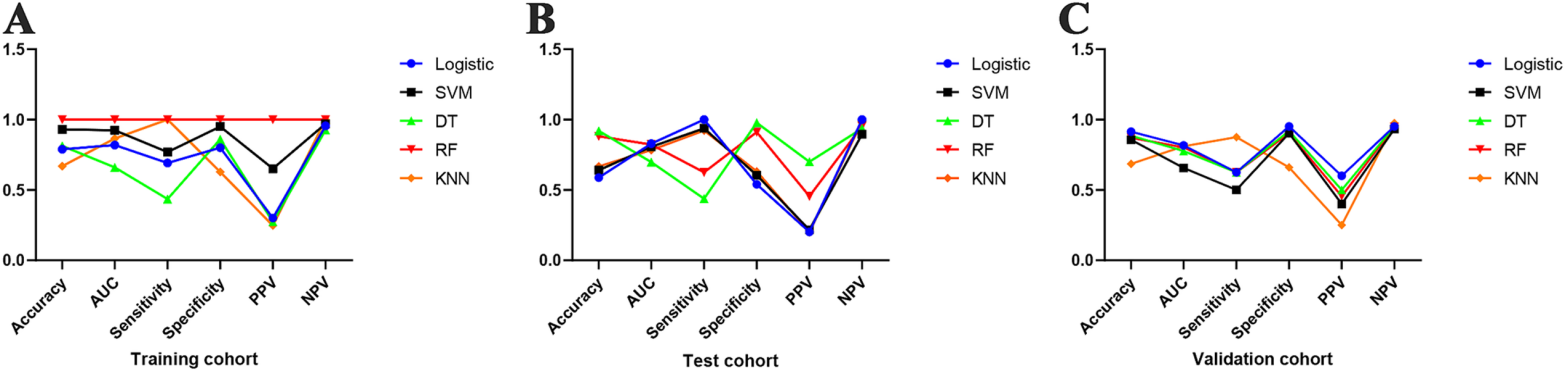
Comparison of the prediction performance of five machine models. **(A)** Line graph comparing the prediction performance of models based on the training cohort; **(B)** line graph comparing the prediction performance of models based on the test cohort; **(C)** line graph comparing the prediction performance of models based on the validation cohort.

We used six indices, namely, accuracy, AUC, sensitivity, specificity, PPV, and NPV, to evaluate the above five GIB models (Table 3). Compared with the other four machine prediction models, the RF model had better accuracy, AUC, sensitivity, specificity, PPV, and NPV in the training cohort (Figure 8A). The KNN model had a higher sensitivity of 1.000, whereas the DT model had a lower sensitivity of only 0.436. The PPVs of all three prediction models, i.e., LR, DT, and KNN, were low. In the test cohort (Figure 8B), the RF model sensitivity and PPV were significantly lower than those in the training cohort, with values of 0.625 and 0.455, respectively, which were intermediate compared with those of the other models. The three models, LR, SVM, and KNN, are much larger than the DT model in terms of sensitivity, but the trend reverses in terms of specificity and PPV. In the validation cohort (Figure 8C), the RF model had approximately the same situation as the test group for each metric. Compared with the other models, KNN had the highest sensitivity (0.875) and the lowest specificity and PPV (0.661 and 0.250, respectively).

**Table 3:** A comparison of gastrointestinal bleeding models’ prediction ability.

Characteristics	LR	SVM	DT	RF	KNN
Training cohort					
Accuracy	0.789	0.931	0.813	1.000	0.669
AUC	0.819	0.924	0.661	1.000	0.865
Sensitivity	0.692	0.769	0.436	1.000	1.000
Specificity	0.801	0.950	0.86	1.000	0.628
PPV	0.300	0.652	0.274	1.000	0.247
NPV	0.955	0.971	0.926	1.000	1.000
Test cohort					
Accuracy	0.588	0.640	0.921	0.882	0.667
AUC	0.829	0.807	0.698	0.823	0.784
Sensitivity	1.000	0.938	0.438	0.625	0.923
Specificity	0.540	0.606	0.978	0.912	0.632
PPV	0.202	0.217	0.700	0.455	0.200
NPV	1.000	0.895	0.937	0.954	0.989
Validation cohort					
Accuracy	0.914	0.857	0.886	0.871	0.686
AUC	0.817	0.657	0.777	0.803	0.811
Sensitivity	0.625	0.500	0.625	0.625	0.875
Specificity	0.952	0.903	0.919	0.903	0.661
PPV	0.600	0.400	0.500	0.455	0.250
NPV	0.952	0.933	0.950	0.949	0.976

LR, logistic regression; SVM, support vector machine; DT, decision tree; RF, random forest; KNN, K-nearest neighbors; AUC, area under the curve; PPV, positive predictive value; NPV, negative predictive value.

## 4 Discussion

GIB is a relatively common complication in patients with HBV-ACLF (24), but whether it affects patient prognosis remains controversial. Studies have reported that upper GIB does not significantly increase the risk of death in patients with ACLF (7). Another study from China explored the effects of artificial liver therapy on the prognosis and survival of patients with ACLF and reported that upper GIB was an independent risk factor for the prognosis of patients with ACLF (5). Trebicka et al. reported that the presence of ACLF was independently associated with rebleeding and mortality in patients with acute variceal bleeding (25). Shin et al. reported a 28-day mortality rate of 41% in patients with variceal bleeding combined with ACLF (26). Our study revealed that among the training cohort of 360 patients, the 30- and 90-day survival rates were significantly lower in the GIB group (48.72 and 10.26%, respectively) than in the NGIB group (85.67 and 64.80%, respectively). Relevant studies have reported that red cell distribution width, platelet count, pulse rate, and SpO2 are risk factors for early mortality in patients with acute upper gastrointestinal bleeding (27). Therefore, early monitoring of these indicators may improve the survival rate of gastrointestinal bleeding patients. ACLF and GIB interact with each other. Decreased coagulation and elevated hepatic venous pressure in patients with ACLF increase the risk of gastrointestinal hemorrhage and increase the difficulty of pharmacological and endoscopic hemostasis; conversely, ischemia and hypoxia after gastrointestinal hemorrhage exacerbate hepatic failure (4). Most studies have focused on the occurrence of ACLF in patients with chronic liver disease induced by GIB (28, 29), and few studies have investigated the prognostic impact of GIB in patients with ACLF, especially HBV-ACLF. Therefore, this study assessed the impact of GIB on the prognosis of patients with HBV-ACLF and constructed a relevant prediction model using ML algorithms to intervene in advance of bleeding events and reduce patient mortality.

The incidence of GIB in patients with HBV-ACLF was 10.8% in this study, which is similar to the 13.86% reported in the relevant literature (6). Seven clinical features associated with GIB were then identified via LASSO regression. The above features were incorporated into five ML algorithms to construct predictive models, and white blood cell count, portal hypertension, and spontaneous bacterial peritonitis were found to be three modeling features common to all five models. White blood cells are inflammatory markers, and their changes can respond to the inflammatory and immune states of the body. Tomizawa et al. studied the risk factors for the development of GIB in 1,023 patients who underwent endoscopy and reported that WBC counts were significantly greater in patients with GIB (30). Another study suggested that elevated white blood cell counts may be associated with nonvariceal upper GIB and its severity (31). White blood cells are significantly more common in patients with GIB who have nonvariceal bleeding than in those with variceal bleeding (32). Patients with HBV-ACLF have elevated white blood cell counts, which may be combined with infections and an increased inflammatory response, which puts the body in a state of stress, as well as causing poor coagulation, ultimately leading to the development of nonvariceal GIB.

Portal hypertension is common among patients with cirrhosis, in which elevated portal venous pressure leads to the obstruction of portal venous blood flow and the formation of collateral circulation in the fundic esophageal vein and other branches, and bleeding from fundic esophageal varices is a common and life-threatening complication (33). A Chinese study in which magnetic resonance imaging was used to construct a predictive model for esophageal variceal bleeding in patients with hepatitis B cirrhosis revealed that portal vein diameter was an independent risk factor for bleeding events (34). Patients with HBV-ACLF combined with decompensated cirrhosis are at high risk of bleeding from fundic esophageal varices and are prone to bleeding events in the presence of coagulation factor deficiency. Electrolyte disturbances are a regular complication among patients with ACLF. Patients have poor appetite, low intake, and excessive loss of electrolytes when ascites occurs, leading to hyponatremia, hypokalemia, etc., which affects patient prognosis (35). Several common electrolytes are involved in coagulation either as coagulation factors themselves or indirectly by regulating the coagulation process. Relevant studies have reported that sodium ions can participate in the coagulation process by binding to thrombin to modulate its conformation, increase thrombin activity, and promote the coagulation cascade reaction (36, 37). Calcium ions, as coagulation IV factors, are involved in coagulation factor activation and platelet function (38). The presence of multiple potassium channels in platelets, such as the Kv1.3, KCa3.1, GIRK and KCa1.1 channels, contributes to a variety of platelet functional responses, such as adhesion and procoagulant activity (39). When electrolyte disturbances occur in patients with HBV-ACLF, imbalances in multiple ion levels may exacerbate patients’ coagulation dysfunction and increase the probability of GIB by affecting platelet or coagulation factor function. Additionally, low total cholesterol levels can increase the fragility of red blood cells and endothelial cells, leading to thinning of the vessel walls and reduced elasticity. These changes make the blood vessels more prone to rupture and bleeding (40). The number of artificial liver treatments indirectly reflects the severity of the patient’s liver failure. Patients who require more frequent treatments generally have more severe conditions, with more pronounced systemic inflammatory responses and unstable hemodynamics, making them more susceptible to GIB (41). Spontaneous bacterial peritonitis triggers a systemic inflammatory response that leads to vasodilation of the visceral vessels, further exacerbating portal hypertension. In addition, inflammatory factors disrupt the coagulation balance and may ultimately directly damage the mucosal barrier, increasing the risk of GIB (42). In patients with HRS, inadequate effective blood volume activates the renin-angiotensin-aldosterone system, further increasing portal pressure, thereby increasing the risk of GIB (43).

With the above seven key clinical features, we constructed prediction models using each of the five ML methods: LR, SVM, DT, RF, and KNN. The model was evaluated via six metrics: accuracy, AUC, sensitivity, specificity, PPV, and NPV. Owing to the uneven sample size between the GIB and NGIB groups of the dataset, it was better to evaluate the model using the AUC as opposed to its accuracy. In the three cohorts, the LR model achieved better AUCs of 0.819, 0.829, and 0.817, respectively, but its PPVs in the modeling cohort and the training cohort were low, 0.300 and 0.202, respectively, which implies that it predicted a preponderance of positive false-positives, a common phenomenon caused by the unequal distribution of the dichotomous variables (44). We then used the model to construct a nomogram that better fit reality and clinical net benefit in the training cohort on the basis of the calibration curve and decision curve results. The RF model outperformed the other four models in the training cohort, with values of 1.000 for all the evaluation metrics. The sensitivity decreased to 0.625, and the PPV decreased to 0.455 in the test cohort, but remained significantly higher than those of the LR, SVM, and KNN models. The RF model significantly improved the PPV while maintaining a better AUC and achieved a similar performance in the validation cohort, with a better overall predictive ability than the other four models. This is related to the fact that the RF model itself is good at handling complex data, reducing overfitting, high accuracy, and robustness. This conclusion was corroborated by a predictive modeling study on omental metastasis of gastric cancer by Ahao et al. (45). The predictive power of the SVM model appeared average in comparison to the other four models, and it had the lowest AUC and sensitivity in the validation cohort at 0.657 and 0.500, respectively. The DT model performed inconsistently in terms of its predictive ability, with a lower AUC and sensitivity than the other four models in both the test and training cohorts. The predictive efficacy of the KNN model remained stable across the 3 cohorts, but the PPV was almost at the bottom, affecting the overall predictive ability of the model. The results of the lower predictive performance of the three models DT, SVM, and KNN may be related to their inherent susceptibility to overfitting, high computational complexity, and sensitivity to feature scaling relative to the RF models (44).

In this study, a prediction model for HBV-ACLF complicating GIB was constructed via five ML methods on the basis of data from two medical centers. The model was subsequently validated internally and externally, and the model similarly demonstrated a high degree of accuracy, providing a decision-making basis for preventing GIB events in ACLF patients in advance for clinical practice. There are several limitations to our study. First, both centers are in the same region, which can further collect patients nationwide and reduce the errors caused by population differences. Second, the sample size of the model constructed in this study is average, and a larger sample size is needed to verify the stability of the model. Finally, this study did not provide detailed information on the types and causes of gastrointestinal bleeding in the patients, nor did it further analyze their impact on patient survival. Future research could address these gaps.

## 5 Conclusion

We found that the short-term survival rate of patients with HBV-ACLF complicated by GIB was significantly lower than that of patients without GIB. On this basis, we constructed five ML models for predicting GIB and found that, compared with the LR, SVM, DT, and KNN models, the RF models have greater comprehensive predictive ability and are more instructive for clinical work.

## Data Availability

The original contributions presented in the study are included in the article/supplementary material, further inquiries can be directed to the corresponding author.
